# Nutcracker syndrome (a Delphi consensus)

**DOI:** 10.1016/j.jvsv.2024.101970

**Published:** 2024-10-02

**Authors:** Floor Heilijgers, Peter Gloviczki, Gerry O'Sullivan, Bertrand Chavent, Efthymios D. Avgerinos, Karem Harth, Stephen A. Black, Young M. Erben, Joris I. Rotmans, Toby Richards, Rabih A. Chaer, Laurencia Villalba, Arjun Jayaraj, Rafael D. Malgor, Ramesh K. Tripathi, Anahita Dua, Erin Murphy, Simon Rinckenbach, Suresh Vedantham, Jaap F. Hamming, Joost R. van der Vorst

**Affiliations:** a Department of Vascular Surgery, Leiden University Medical Center, Leiden, the Netherlands; b Division of Vascular and Endovascular Surgery, Mayo Clinic, Rochester, MN; c Department of Interventional Radiology, Galway University Hospital, Galway, Ireland; d Department of Vascular Surgery, Clinique Générale, Annecy, France; e Department of Vascular Surgery, Athens Medical Center, University of Athens, Athens, Greece; f Department of Vascular Surgery, University Hospitals Cleveland Medical Center, Case Western Reserve, Cleveland, OH; g Department of Vascular Surgery, Ashtead Hospital, Ashtead, UK; h Division of Vascular and Endovascular Surgery, Mayo Clinic, Jacksonville, FL; i Department of Internal Medicine, Leiden University Medical Center, Leiden, the Netherlands; j Department of Anesthesia and Perioperative Medicine, Monash University, Melbourne, Victoria, Australia; k Department of Surgery, School of Health, Sport & Bioscience University of East London, London, UK; l Institute of Clinic Trials and Methodology, University College London, London, UK; m Department of Vascular Surgery, Wollongong Hospital, Wollongong, New South Wales, Australia; n Department of Vascular Surgery, The Rane Center for Venous and Lymphatic Disorders, Jackson, MS; o Department of Vascular Surgery, University of Colorado Anschutz Medical Center, Aurora, CO; p Department of Vascular Surgery, University of Queensland, Brisbane, Queensland, Australia; q Department of Vascular Surgery, Massachusetts General Hospital and Harvard University, Boston, MA; r Department of Vascular Surgery, Atrium Health Sanger Heart and Vascular Institute, Charlotte, NC; s Department of Vascular and Endovascular Surgery, University of Franche Comté, Besançon, France; t Mallinckrodt Institute of Radiology, Washington University School of Medicine, St. Louis, MO

**Keywords:** Nutcracker Syndrome, NCS Delphi consensus Left Renal Vein, LRV LRV transposition Endovascular Stenting, EVS Renal Autotransplantation

## Abstract

**Background:**

Nutcracker syndrome (NCS) describes the symptomatic compression of the left renal vein between the aorta and superior mesenteric artery. Whereas asymptomatic compression is a common radiological finding, patients with NCS can report a range of symptoms. There are no specific diagnostic criteria and interventions include a range of open surgical and endovascular procedures. Therefore, we wished to develop an international consensus document covering aspects of diagnosis, management, and follow-up for patients with NCS.

**Methods:**

A three-stage modified Delphi consensus was performed. A steering committee developed 37 statements covering 3 categories for patients with NCS: diagnosis, management, and follow-up. These statements were reported individually by 20 international experts in the management of venous disease, using a 5-point Likert scale. Consensus was defined if ≥70% of respondents rated the statement between 1 and 2 (agreement) and between 4 and 5 (disagreement). Those statements without consensus were recirculated in a second round of voting. A third round of the questionnaire was performed with 14 additional statements to clarify diagnostic values of NCS.

**Results:**

Responses were returned by 20 of 20 experts (100%) in round one and 17 of 20 (85%) in round two. Initial consensus was reached in 24 of 37 statements (65%) spread over all categories. Round two achieved a further consensus on 5 out of 10 statements (50%). No categories reported consensus on all statements. In round two consensus was reached in the category of follow-up (4/5 statements [80%]). The final round reached consensus on 5 out of 14 statements (36%). Experts agreed that imaging is obligated to confirm NCS. Experts did not agree on specific diagnostic cut-off values. There was a consensus that the first choice of operative treatment is left renal vein transposition and that the risk of stent migration outweighs the advantages of a percutaneous procedure.

**Conclusions:**

Consensus was achieved on most statements concerning the assessment and management of NCS. This Delphi consensus identified those areas in which further research is needed, such as antiplatelet therapy, endovascular treatment, and renal autotransplantation. A rare disease registry to improve data and reports of patient outcomes is warranted.

Nutcracker syndrome (NCS) describes the symptomatic compression of the left renal vein (LRV)^1^ between the aorta and superior mesenteric artery. The term nutcracker phenomenon is often used for the anatomical finding of LRV compression when there are no clinical symptoms present.^2^ Whereas asymptomatic compression is a common radiological finding, patients with NCS can report a range of symptoms. Reduction of venous outflow from the left kidney through the LRV into the inferior vena cava can lead to renovenous hypertension. This in turn can lead to venous collateral development or incompetence and retrograde flowing in the ancillary veins and subsequent secondary affect in the pelvis and/or lower limbs. Other forms of NCS include posterior NCS, in which the retroaortic renal vein may be compressed between aorta and the spine.^3^^,^^4^

Patients with NCS can present with a variety of symptoms, including hematuria, proteinuria, and left flank and pelvic pain.^5^ NCS can be challenging to diagnose, owing to the variety of symptoms and because no specific diagnostic criteria have been validated. NCS can often be a diagnosis by exclusion of other causes. Most diagnostic modalities including computed tomography venography, magnetic resonance venography, and duplex ultrasound examination have been described as well as invasive phlebography, venous pressure measurement and intravascular ultrasound examination.

Whereas incidental findings of venous compression are common,^6^ conversely the prevalence of NCS is unknown and may be slightly higher in female patients.^2^^,^^3^^,^^5^^,^^6^ Interventional treatments of NCS includes both open surgical and endovascular interventions, which can focus on the LRV itself or decompression of the venous outflow from the kidney elsewhere.^1^

The absence of evidence concerning NCS makes it difficult to make recommendations on the diagnostics and management of this condition. Therefore, the objective of this study was to reach a Delphi consensus on the diagnostics, treatment, and follow-up for patients with NCS using an international, multispecialty expert panel. An additional goal was to identify areas where consensus was lacking and focus future clinical research on those fields.

## Methods

### Delphi procedure

The Delphi technique is a survey used to methodically gather judgement from an expert panel, which prevents one expert from dictating consensus.^7^, ^8^, ^9^ This method allows researchers to include a number of participants throughout the world. It relies on experts anonymously answering questionnaires in two or more rounds. During this process, it is anticipated that the range of answers will decrease, and the group will converge toward a consensus answer. The process continues until participants reach consensus or if no additional consensus is expected. The anonymity removes inherent biases like dominance and group conformity. Furthermore, the switch between favorable and unfavorable worded statements decreases risk of agreement bias. This study consisted of a three-round Delphi consensus process and took place between September 2023 and June 2024.

### Expert panel

International multispecialty experts^7^ was identified from lists of venous network contacts from the study authors or from recent publications on NCS.^1^, ^2^, ^3^^,^^5^ A total of 20 international experts were selected based on their specialties and reports on NCS, who represented a broad geographic distribution.

### Delphi questionnaire

Two authors (F.H. and J.R.V.) designed the initial questionnaire and were responsible for collecting and organizing data and communicating with the expert panel. A steering committee consisting of seven experts evaluated and approved the final version of the questionnaire.

The initial questionnaire consisted of 37 statements that were divided in to 3 categories to allow thematic grouping: diagnosis, management, and follow-up. To indicate the level of agreement or disagreement, survey participants were asked to rate each statement on a 5-point Likert scale, with a rating of 1 indicating strong agreement and 5 indicating strong disagreement. Experts could leave comments after each statement to clarify their agreement or disagreement or for suggestions to adjust the statement in the next round.^10^ Consensus was defined as ≥70% of received scores between 1 and 2 (indicating agreement with the statement) or between 4 and 5 (indicating disagreement with the statement).^6^ In this study, the questionnaire was developed using the web-based platform Formdesk (Formdesk, Wassenaar, the Netherlands) and the invitations to participate were sent out via email in September 2023, with reminder emails sent to nonresponders. The first round of the questionnaire was sent out in October 2023 (Appendix A).

#### Revision of statements

**Fig 1:**
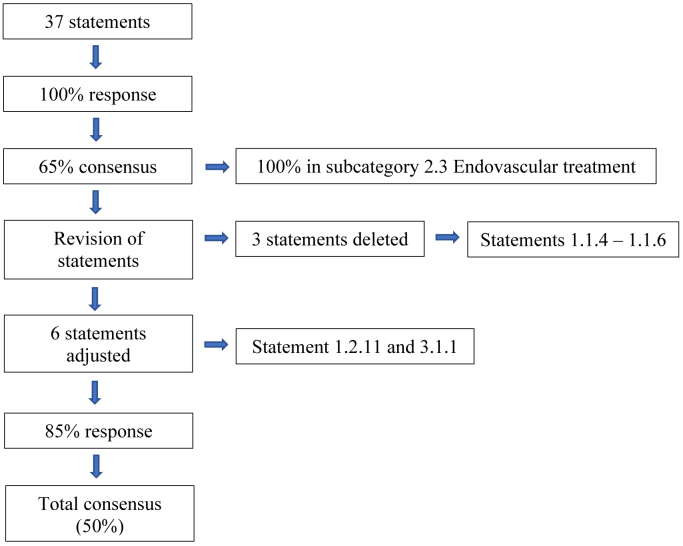
Flowchart of the Delphi consensus method.

Results of round one (with free text comments) were reviewed by the authors (F.H. and J.R.V.). The statements that did not reach consensus in round one were reviewed and if the statement was perceived by the experts as ambiguous minor revisions were incorporated. Round two consisted of only the statements that did not reach consensus in the first round, including the option to leave comments after each statement. This is shown in Fig 1. The same categories were used (Appendix B). Invitation to round two were sent to the original 20 experts through the same process in October 2023, with reminders sent to nonresponders. To clarify diagnostic values concerning NCS, a third round of the questionnaire was sent out in June 2024 containing fourteen additional statements with comment sections (Appendix C). The survey was closed in June 2024. To reduce agreement bias we added negative statements to the survey. The concept remained unclear to our panel given the comments that were made, which is why we chose to remove statement 1.1.6 from the questionnaire. Furthermore, panelists voted neither agree nor disagree on statements that were not based on recent (randomized of prospective) literature, because evidence is lacking owing to the rarity of NCS. This made it difficult to reach consensus on statement 2.2.3, for instance.

## Results

Table I shows the demographic characteristics of the expert panel. Round one of the Delphi questionnaire received 20 of 20 complete responses (100%) from the identified expert panel and reached consensus on 24 out of the 37 statements (65%). Among the statements on which consensus was reached, consensus reached from 70% to 100%.

**Table I:** Demographic characteristics of expert panel

(Sub)specialty	
Vascular surgery	17 (85)
Interventional radiology	2 (10)
Internal medicine	1 (5)
Affiliation	
Country	
North and South America	10 (50)
Europe	8 (40)
Asia Pacific	2 (10)
Type of medical centre	
Tertiary or academic	19 (95)
Private	1 (5)
Years of experience	17 (6-52)
No. of patients in outpatient clinic yearly	21 (3-80)
No. of patients treated yearly	6 (1-15)

Data are number (%) or mean (range).

The results of round one of the Delphi are summarized in Table II and reported consensus on all statements in subcategory 2.3 Endovascular treatment (3/3 statements [100%]). In the other (sub)categories consensus was reached in symptoms and clinical features (4/7 statements [57%]); diagnosis (imaging) (10/12 statements [83%]); conservative treatment (2/4 statements [50%]); and operative treatment (5/6 statements [83%]). Category 3 Follow-up did not reach consensus on the statements. Three statements were deleted from the questionnaire after the round one. Comments received on the statements that were adjusted or deleted after the round one are shown in the Supplementary Table (online only).

**Table II:** Round 1 Delphi statements results

Statements	Strongly agree	Agree	Neither agree nor disagree	Disagree	Strongly disagree	Consensus^a^
1. Diagnosis						
1.1 Symptoms and clinical features						
1.1.1 NCS is a combination of clinical signs and symptoms caused by stenosis of the LRV.	10	10	0	0	0	Yes (100)
1.1.2 The characteristic clinical signs and symptoms of NCS include hematuria and flank pain.	8	12	0	0	0	Yes (100)
1.1.3 The discovery of asymptomatic compression of the LRV is called nutcracker phenomenon.	0	17	1	1	1	Yes (85)
1.1.4 Hematuria specific to NCS is caused by rupture of the thin-walled septum between small veins and collecting system of renal fornix.	1	11	8	0	0	No (60)
1.1.5 Proteinuria specific to NCS is caused by increased pressure in the LRV, which leads to the release of angiotensin II and norepinephrine.	3	6	11	0	0	No (45)
1.1.6 NCS is not related to pelvic venous disorders.	0	3	4	9	4	No (15)
1.1.7 Pelvic venous disorders can be caused by NCS.	6	12	1	1	0	Yes (90)
1.2 Diagnostics						
1.2.1 Imaging is obligated to confirm NCS.	16	4	0	0	0	Yes (100)
1.2.2 DUS should be used in the diagnostic workup of NCS patients.	9	9	1	1	0	Yes (90)
1.2.3 Because of the highly variability index and range of values of DUS in NCS, it should not be used as a single diagnostic modality to diagnose NCS.	12	8	0	0	0	Yes (100)
1.2.4 DUS in NCS patients should at least describe the peak systolic velocity at stenosis and in the distal renal vein and the diameter of the LRV at the stenosis and distal to the stenosis.	5	13	1	1	0	Yes (90)
1.2.5 Venous CT or MRI can demonstrate compression of the LRV between the SMA and the aorta or between the aorta and the spina.	5	15	0	0	0	Yes (100)
1.2.6 CT or MRI do not have a role in the diagnostic workup in NCS patients.	0	0	2	9	9	Yes (90)
1.2.7 CT or MRI should always be performed before surgery to exclude other potential causes of symptoms and for accurate surgical planning.	10	10	0	0	0	Yes (100)
1.2.8 Contrast phlebography is considered the gold standard for diagnosis of NCS.	3	5	5	7	0	No (40)
1.2.9 Venous pressure measurements and observation of collateral flow are the most useful phlebographic signs of significant LRV obstruction.	3	11	4	2	0	Yes (70)
1.2.10 The absence of a significant pressure gradient does not exclude NCS.	3	12	4	1	0	Yes (75)
1.2.11 A significant pressure gradient is defined as >2 mm Hg.	2	11	6	1	0	No (65)
1.2.12 Intravascular ultrasound can be used to assess the severity of LRV stenosis in NCS patients.	7	11	2	0	0	Yes (90)
2 Management						
2.1 Conservative treatment						
2.1.1 A conservative approach should be the first treatment option in all NCS patients.	8	8	0	2	2	Yes (80)
2.1.2 Conservative treatment with an emphasis of weight gain should be the first step in treating all NCS patients with a low body weight (BMI of <18.5 kg/m^2^).	4	12	4	0	0	Yes (80)
2.1.3 Aspirin should be considered if the patient with NCS shows sign of kidney failure to improve renal perfusion.	0	1	9	8	2	No (50)
2.1.4 Aspirin should be prescribed when diagnosing NCS.	0	0	9	9	2	No (55)
2.2 Operative treatment						
2.2.1 First choice of operative treatment is LRV transposition.	3	13	2	2	0	Yes (80)
2.2.2 If successful LRV transposition does not lead to symptom release, the diagnosis NCS should be reconsidered.	2	12	3	2	1	Yes (70)
2.2.3 Renal autotransplantation is an effective procedure.	1	10	8	1	0	No (55)
2.2.4 LGV transposition can be an alternative for LRV transposition in selected patients.	4	13	2	1	0	Yes (85)
2.2.5 LGV transposition can be performed into the left iliac vein in patients who do not have May-Thurner syndrome.	4	13	2	1	0	Yes (85)
2.2.6 Too little evidence is present to have a preference for laparoscopic or open surgical procedures.	5	10	4	1	0	Yes (75)
2.3 Endovascular treatment						
2.3.1 The risk of stent migration outweighs the advantages of a percutaneous procedure, thus stenting is not recommended as a primary treatment for NCS.	9	5	1	5	0	Yes (70)
2.3.2 With an unknown long-term outcome of renal vein stents, open interventions are a safer option for NCS.	9	8	2	1	0	Yes (85)
2.3.3 EVS is the preferred treatment over open procedures.	0	0	4	7	9	Yes (80)
3 Follow-up						
3.1 Anticoagulation of antiplatelet medication						
3.1.1 After LRV transposition, patients should be treated with platelet aggregation inhibitors for the total duration of ≥6 months.	3	9	1	6	1	No (60)
3.1.2 Patients must be followed up using DUS yearly.	2	11	5	2	0	No (65)
3.1.3 First moment of follow-up should be before 6 weeks.	4	9	4	3	0	No (65)
3.1.4 Patients with NCS should be followed up yearly.	1	10	7	1	1	No (55)
3.1.5 Follow-up can be ended after a period of 2 years.	1	1	7	9	2	No (55)

*BMI,* Body mass index; *CT,* computed tomography; *DUS,* duplex ultrasound; *EVS,* endovascular stenting; *LGV,* left gonadal vein; *LRV,* left renal vein; *MRI,* magnetic resonance imaging; *NCS,* nutcracker syndrome; *SMA,* superior mesenteric artery.

a Consensus is defined as ≥70%. The highest percentage (agree and strongly agree divided by total or disagree + strongly disagree) divided by total) is presented.

The second round of the Delphi questionnaire received 17 of 20 responses (85%) from the identified expert panel and reached consensus on 5 of the 10 statements (50%). The results of the second round are summarized in Table III. In none of the categories was consensus reached on all statements. Consensus was reached in category Follow-up (4/5 statements [80%]). Furthermore, there was a consensus that the first choice of operative treatment is LRV transposition and that the risk of stent migration outweighs the advantages of a percutaneous procedure.

**Table III:** Round 2 Delphi statements results

Statements	Strongly agree	Agree	Neither agree nor disagree	Disagree	Strongly disagree	Consensus^a^
1. Diagnosis						
1.2 Diagnostics						
1.2.8 Contrast phlebography is considered the gold standard for diagnosis of NCS.	4	3	0	9	1	No (59)
1.2.11 A significant mean pressure gradient is defined as >2 mm Hg in a supine position.	4	6	4	3	0	No (59)
2. Management						
2.1 Conservative treatment						
2.1.3 Aspirin should be considered if the patient with NCS shows sign of kidney failure to improve renal perfusion.	1	1	5	6	4	No (59)
2.1.4 Aspirin should be prescribed when diagnosing NCS.	0	3	4	5	5	No (59)
2.2 Operative treatment						
2.2.3 Renal autotransplantation is an effective procedure.	4	7	5	1	0	No (65
3 Follow-up						
3.1 Anticoagulation or antiplatelet medication						
3.1.1 After LRV transposition patients should be treated with some form of platelet aggregation or anticoagulation.	8	7	1	1	0	Yes (88)
3.1.2 Patients can be followed using DUS yearly in long-term follow-up.	7	10	0	0	0	Yes (100)
3.1.3 First moment of imaging (CT scan or DUS) after surgery should be before 6 weeks.	7	7	0	2	1	Yes (82)
3.1.4 Patients with NCS who underwent surgery should be followed up yearly.	7	9	1	0	0	Yes (94)
3.1.5 Follow-up after surgery can be ended after a period of 5 years.	2	8	1	5	1	No (59)

*CT,* Computed tomography; *DUS,* duplex ultrasound; *LRV,* left renal vein; *MRI,* magnetic resonance imaging; *NCS,* nutcracker syndrome.

a Greater than or equal to 70%, with the highest percentage of (dis)agreement.

**Fig 2:**
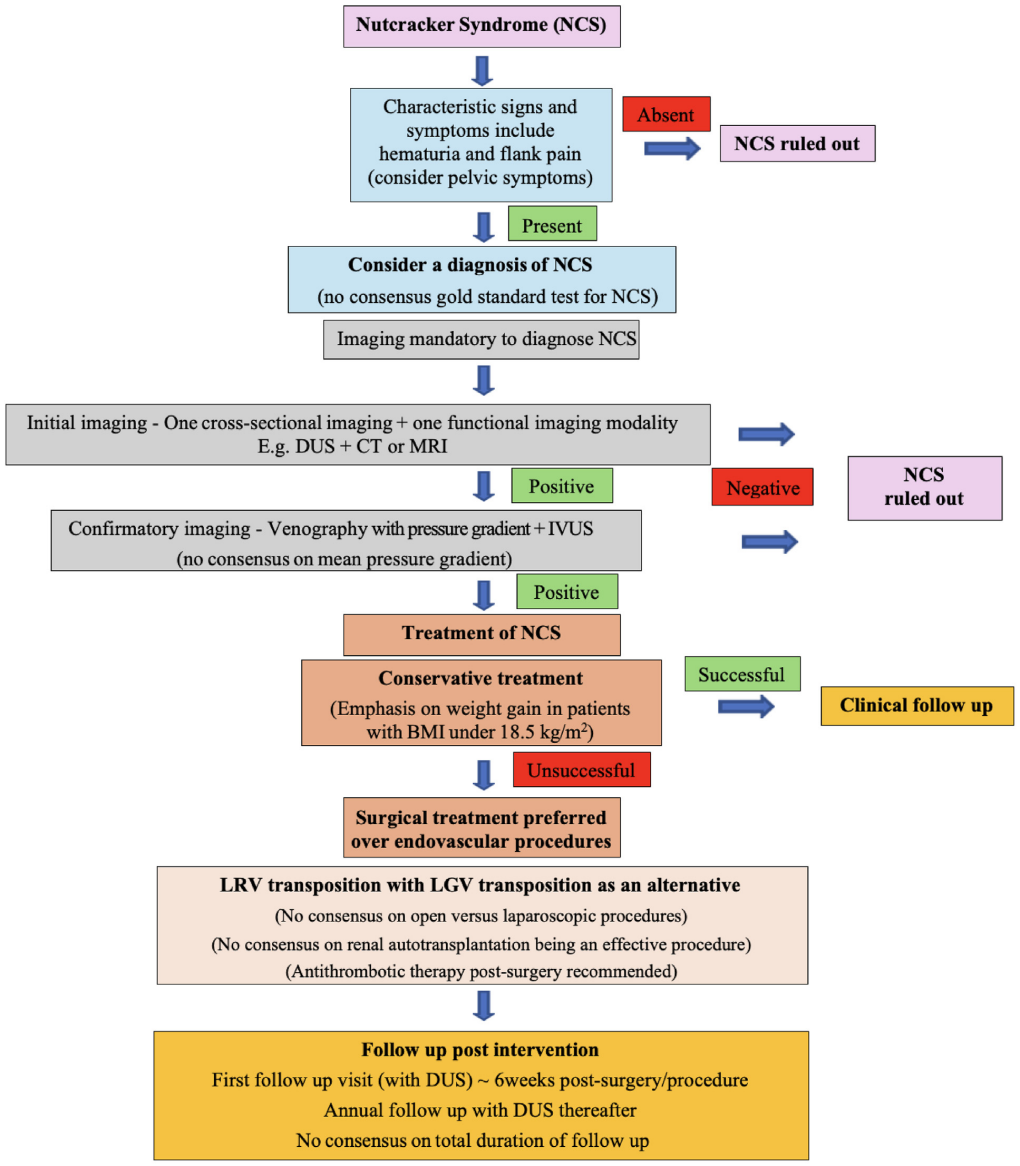
Flowchart for the management of nutcracker syndrome (NCS) (Delphi consensus). *CT*, Computed tomography; *DUS*, duplex ultrasound; *IVUS*, intravascular ultrasound; *LGV*, left gonadal vein; *LRV*, left renal vein; *MRI*, magnetic resonance imaging.

The final round of the Delphi questionnaire received 19 of 20 responses (95%) from the identified expert panel and reached consensus on 5 of 14 statements (36%) with one statement reaching nearly consensus with 68%. The results of this round are summarized in Table IV. The panel agreed that we cannot prove or disprove a form of imaging as a golden standard until we have randomized controlled trials or strong multi-institutional data. There was no consensus the cut-off values in imaging of NCS in the diagnostic process or during follow-up. Experts did agree that phlebography has a role in the diagnostic process of NCS, combined with some form of cross-sectional imaging. The results of this Delphi consensus method are shown in Fig 2.

**Table IV:** Round 3 Delphi statements results

Statements	Strongly agree	Agree	Neither agree nor disagree	Disagree	Strongly disagree	Consensus^a^
1 Diagnosis						
1.1 Symptoms and clinical						
1.1.13 The compressive process causes varying levels of extrinsic stenosis of the renal branch, which results in asymptomatic episodes alternated with symptomatic episodes.	2	8	5	2	1	No (53)
1.1.14 Classify these signs and symptoms concerning NCS from most to least relevant: flank pain, hematuria, proteinuria, pelvic pain.	-	-	-	-	-	1 Flank pain 2 Hematuria 3 Pelvic pain 4 Proteinuria
1.1.15 In case of NCS, signs and symptoms (such as hematuria and flank pain) must last >6 months.	6	8	3	2	0	Yes (74)
1.2 Diagnostics						
1.2.13 There is no gold standard for imaging used in diagnosing NCS.	4	10	1	4	0	Yes (74)
1.2.14 The percentage of stenosis of LRV is a useful factor and >50% stenosis measured by DUS should be considered significant.	0	7	3	6	4	No (53)
1.2.15 A distance between the SMA and aorta of <8 mm is considered abnormal.	2	6	10	1	0	No (42)
1.2.16 During ultrasound, a ratio of >4 between the diameter of the hilar renal vein and the diameter of the renal vein at the aortic mesenteric window is considered abnormal.	2	6	8	3	0	No (42)
1.2.17 An aortic – SMA angle of <30° is an abnormal finding.	1	5	8	5	0	No (37)
1.2.18 Phlebography has a place in the diagnostic process, as it provides information about the pressure gradient, collateral veins, flow pattern and LGV.	11	7	0	1	0	Yes (95)
1.2.19 NCS can be excluded without venography.	3	9	1	4	1	No (68)
1.2.20 In case of a BMI of >25 km/m^2^, a diagnosis other than NCS must be considered.	3	4	6	6	0	No (37)
1.2.21 At least one form of cross-sectional imaging associated to one functional imaging modality should be performed in the diagnostic work-up of NCS.	8	9	1	1	0	Yes (89)
3 Follow-up						
3.1 Anticoagulation or antiplatelet medication						
3.1.6 During follow-up, patients with NCS should be checked for stent patency or severity of stenosis in LRV.	10	8	0	1	0	Yes (95)
3.1.7 Greater than fifty percent restenosis in the LRV should lead to reintervention.	1	5	5	7	1	No (43)

*SMA,* superior mesenteric artery; *BMI,* Body mass index; *DUS,* duplex ultrasound; *LGV,* left gonadal vein; *LRV,* left renal vein; *NCS,* nutcracker syndrome; *SMA,* superior mesenteric artery.

a Greater than or equal to 70%, with the highest percentage of (dis)agreement.

## Discussion

In this 3-round Delphi consensus, 20 international venous experts reached consensus on 28 of 37 statements concerning the diagnosis, management and follow-up of NCS. This study demonstrates that despite the lack of high-quality evidence guiding the diagnosis, management and follow-up of NCS, consensus was achieved for most statements in the three rounds of this Delphi process. Much of this consensus is most likely related to recent literature.^1^, ^2^, ^3^^,^^5^ However, many aspects of diagnosis and treatment are still subject to individual variation and experience. This variability was confirmed by the lack of consensus in multiple facets of treatment and follow-up.

### Diagnosis

Consensus was reached on the combination of clinical signs of NCS and stenosis of LRV being a cause of these symptoms. Flank pain was ranked as the most relevant symptom in NCS, it being the main symptom of NCS differentiating it from other causes of pelvic venous disorders. The distinction between NCS and pelvic venous disorders remains a challenge in the diagnostic workup. It is not in the scope of this article to gauge consensus on concomitant NCS and pelvic venous disorders. Further research is needed to standardize the assessment of pelvic venous disorders and NCS.

No consensus was reached on the pathological cause of hematuria and proteinuria in NCS, despite supplying the panel with known theories. The mechanism is unknown. Therefore statements 1.1.4 and 1.1.5 were removed from the survey after the first round. Furthermore, proteinuria was ranked as the least relevant symptom of NCS, because it is most likely to be solely present in a case of kidney injury. It was emphasized that these symptoms individually are nonspecific. Because NCS is not acute pathology, the persistent character should be checked. Our panel agreed that symptoms of NCS should last ≥6 months and that compression should be demonstrated. The mechanism for the asymptomatic episodes alternated with symptomatic episodes in NCS remains unknown owing to a lack of evidence.

The expert panel agreed on the different diagnostic modalities that can be used in diagnosing NCS and that there is no golden standard in the diagnostic process of NCS. Randomized controlled trials and strong multi-institutional data are needed to prove or disprove a form of imaging as golden standard. However, experts did agree that phlebography has a place in the diagnostic process, as it provides information about the presence of collateral veins, flow patterns, status of the left gonadal vein and can be combined with measurement of pressure gradients easily. They felt it should be combined with least one form of cross-sectional imaging. Additional statements regarding cut-off values of imaging modalities did not reach consensus. These values are supportive if there are symptoms that correlate and symptom manifestation is related to a variety of factors. The combination of radiological findings linked to clinical findings is imperative.

### Management

Experts agreed that conservative treatment with an emphasis of weight gain should be the first step in treating all NCS patients with a low body weight (body mass index [BMI] of <18.5 kg/m^2^). However, there was no consensus on a BMI value over which NCS should be excluded. The panel emphasized that NCS is a diagnosis by exclusion and that no matter the BMI, all other diagnosis should be considered.

Notable was the consensus reached in the first round on open procedures for NCS being the preferred treatment. Experts agreed that the advantages of percutaneous procedures did not outweigh the risk of complications of endovascular treatment. As shown in the literature,^9^^,^^11^^,^^12^ stent migration is a recognized complication of endovascular stenting. Most reported cases occurred with shorter and smaller diameter stents. In case of LRV stenting, the unfavorable geometry (combination of short length of the target venous segment and the orthogonal angle from the inferior vena cava) makes accurate and stable large-stent placement challenging. Furthermore, precise placement is challenged by abdominal motion during breathing. The paucity of reported data with often limited follow-up makes it challenging to prove the long-term stability of stent placement confidently. These findings combined with those from Sayed et al (2022)^13^ suggest that stent migration might be under-reported. However, the expert panel acknowledges that dedicated venous stents, better sizing, holding respiration, and intravascular ultrasound-assisted stenting are potential improvements that make stenting in this area safer. Recent studies on stent migration are summarized in Table V.^13^, ^14^, ^15^, ^16^, ^17^, ^18^, ^19^, ^20^, ^21^, ^22^, ^23^, ^24^

**Table V:** Reported stent migration and complications

Study	Year	Cohort (patients)	Stent type and size	Outcomes	Reintervention/Complication	Follow-up, months
Cizman et al	2024	1	8 × 50 mm Viabahn stent (W. L. Gore & Associates)	Stent migration from LRV to right pulmonary artery	Endovascular removal with cardiothoracic surgery back-up	-
Cooley et al	2023	1	9 × 39 mm Abbott Omnilink Elite	Stent migration from LVR to left pulmonary artery 6 months postoperatively	Failed removal of stent with balloon manipulation Successful balloon angioplasty	3
Luo et al	2023	1	10 × 30 mm Abbott Vascular Omnilink Elite	Stent migration from LRV to right ventricle 5 days postoperatively	Endovascular retrieval	-
Sayed et al	2022	52	Data on 49 of 54 stents: 2 Viahban (W. L. Gore & Associates), 1 Abbott Vascular Omnilink Elite, 9 Palmaz (Cordis, Europe), 6 S M A.R.T. Control Stent (Cordis, Europe), 2 Protégé (Medtronic), 1 Symphony (Boston Scientific), 1 Epic (Boston, Scientific), 1 Luminexx (BD Interventional), 1 Zilver (Cook, Medical), 1 Supera (Abbott Vascular) 38 were <60 mm in length (none >100 mm), 3 were ≤14 mm in diameter,	Stent migration to: - Right atrium (9)^a^ - Right ventricle (21)^a^ - Pulmonary artery (11)^a^ - IVC (10)^a^ - SMV (1)^a^ - SVC (1)^a^	Endovascular retrieval in 30 of 46 (65.5%) and open surgery in 16 of 46 (34.7%)	1
Ober et al	2022	1	9 × 40 mm Zeus CC balloon expandable stent (Rontis AG)	Stent migration from LRV to renal side of vein with direct replacement during intervention	None	3
Zhang et al	2021	1	14 × 40 mm nitinol vascular stent (Johnson & Johnson)	Stent migration from LRV to right ventricle, accompanied with tricuspid insufficiency 1 month postoperatively	Stent removal through thoracotomy	0.5
Miller et al	2021	1	14 × 60 mm Vici Venous Stent (Boston Scientific)	Stent migration from subclavian vein to right ventricle 10 days postoperatively	Endoscopic robotic retrieval of stent	1
Sebastian et al	2017	1	14 × 40 mm S M.A.R.T. Control Stent (Cordis Europe)	Stent migration from LRV to right ventricle 2 days postoperatively	Endovascular stent retrieval	3
Wu et al	2016	75	7 Wallstents (Boston Scientific), 68 S M A.R.T. Control Stent (Cordis) Both between 10-14 × 40 mm	5 (6.7)^¥^ cases of stent migration from LRV to: - Right ventricle (1)^a^ - Right atrium (1)^a^ - IVC (2)^a^ - Left side of LRV (1)^a^	Open cardiac surgery with or without tricuspid valve replacement, stent extraction and IVC-LRV bypass	6-126 (55)^b^
Chen et al	2015	1	14 × 40 mm, self-expanding, endovascular stent	Stent migration from LRV to right ventricle 5 months postoperatively	Open cardiac surgery for stent removal with prosthetic valve replacement	-
Tian et al	2015	1	10 × 40 mm Smart Control stent (Cordis, Johnson & Johnson)	Stent migration from LRV to the left of SMA 1 month postoperatively	Extravascular stent placement of LRV	36
Rana et al	2013	1	14 × 20 mm Wallstent (Boston Scientific)	Stent migration from LRV to IVC 1 day postoperatively	Endovascular retrieval of stent and replacement with 18 × 40 mm Wallstent	10

*IVC,* Inferior vena cava; *LRV,* left renal vein; *SMA,* superior mesenteric artery; *SMV,* superior mesenteric vein; *SVC,* superior vena cava.

a Number of events.

b Mean.

The downside of open procedures contains risk of infection and perioperative morbidity, among others. Therefore, further data and endovascular treatment would be desirable.

No consensus was reached on the role of antithrombotic therapy in the treatment of NCS. However, it was emphasized that it is important to maintain patency since occlusion is challenging to treat. Last, no consensus was reached on renal autotransplantation being an effective procedure. This result indicates the rarity of the disease and unfamiliarity of its management and that further research is needed, for example, using a rare disease registry to improve data and report of patient outcomes.

### Follow-up

The lack of consensus was most common in section 3 concerning follow-up. All statements were adjusted according to the comments we received, after which consensus was reached on four of the five statements. Experts agreed that stent patency and degree of stenosis in the LRV are important factors to check during follow-up. However, no consensus was reached on the cut-off value that should lead to reintervention. The panel emphasized that this decision depends on the clinical picture and patient condition.

No consensus was reached on when follow-up can be ended in patients with NCS who underwent surgery. This result shows the importance of knowing the long-term effects after surgery in NCS, which remain unknown owing to the rarity of the disease.

The panelists were identified from lists of venous network contacts from the study authors or from recent publications on NCS. Ten experts did not have recent publications concerning NCS. However, this factor demonstrates that high-quality evidence is still lacking owing to the rarity of this disease. The expert panel in this study was composed of an international and multispecialty group of venous experts. Although this composition could be considered a strength, it could also have contributed to some of the lack of consensus as intrinsic differences between healthcare systems, treatment pathways and equipment availability may have led to different interpretations of statements. The panel consisted of 1 nephrologist, 2 interventional radiologists, and 17 vascular surgeons. The consensus on endovascular strategies could be different with a broader expert panel from a variety of specialties managing venous diseases, such as interventional radiologist. It is conceivable that with a different mix of interventionalists, an alternate consensus on treatment options may have been arrived at. The nonsystematic method of selecting panel members may also have had an effect on the outcome of this Delphi project. Participants were primarily from private tertiary or academic institutions, which also provides a different experience. Such a distribution reflects the rarity of NCS because, as with most rare conditions, treatment occurs in private tertiary or academic practices. We chose to include an expert panel mostly working in academic settings as they are able to offer both open surgery and stent-based procedures. It is possible that, owing to this selection method, high-volume specialists practicing in hospital-employed realms were not included in our panel, which can be seen as a limitation.^10^ Another limitation concerning our Delphi may be the size of the expert panel (20 panelist in the first round, 17 panelists in the second round, and 19 panelists in the third round), which is smaller than recent Delphi consensus research.^6^^,^^25^ However, our panel size was comparable to a Delphi study by de Mik et al (2019).^8^ This project has identified those areas in which further research should be focused, for example using international patient registries. These areas are shown in Table VI.

**Table VI:** Areas to focus on in future studies

Diagnosis
Etiology of hematuria and proteinuria
Gold standard for diagnosis
Internal Medicine
Management
Role of aspirin
Renal autotransplantation
Endovascular treatment
Follow-up
Duration

## Conclusions

This study identified areas of consensus among selected vascular experts regarding the assessment and management of NCS. Areas where consensus was not evident reflected the rarity of the disease, challenging diagnosis, and unknown long-term effects of treatment. This Delphi consensus stresses that caution should be applied on treating patients with NCS with endovascular techniques because of the potential of stent migration and thrombosis. Further studies are needed in several areas including using a rare disease registry to improve data and report of patient outcomes.

## Author Contributions

Conception and design: FH, PG, GO, BC, EA, KH, SB, YE, JR, TR, RC, LV, AJ, RM, RT, AD, EM, SR, SV, JH, JVDV

Analysis and interpretation: FH, PG, JH, JVDV

Data collection: FH, PG, GO, BC, EA, KH, SB, YE, JR, TR, RC, LV, AJ, RM, RT, AD, EM, SR, SV, JH, JVDV

Writing the article: FH, JVDV

Critical revision of the article: FH, PG, GO, BC, EA, KH, SB, YE, JR, TR, RC, LV, AJ, RM, RT, AD, EM, SR, SV, JH, JVDV

Final approval of the article: FH, PG, GO, BC, EA, KH, SB, YE, JR, TR, RC, LV, AJ, RM, RT, AD, EM, SR, SV, JH, JVDV

Statistical analysis: Not applicable

Obtained funding: Not applicable

Overall responsibility: JVDV

## Disclosures

None.

## Appendix (online only)

Supplementary Table I (online only)

Appendices

## References

[1] Dieleman F., Hamming J.F., Erben Y., van der Vorst J.R. (2023). Nutcracker syndrome: challenges in diagnosis and surgical treatment. Ann Vasc Surg.

[2] Buschi A., Harrison R., Norman A. (1980). Distended left renal vein: CT/Sonographic normal variant. Am J Roentgenol.

[3] Nastasi D.R., Fraser A.R., Williams A.B., Bhamidi V. (2022). A systematic review on Nutcracker Syndrome and proposed diagnostic algorithm. J Vasc Surg Venous Lymphat Disord.

[4] Ananthan K., Onida S., Davies A.H. (2017). Nutcracker syndrome: an update on current diagnostic criteria and management guidelines. Eur J Vasc Endovasc Surg.

[5] Orczyk K., Wysiadecki G., Majos A., Stefańczyk L., Topol M., Polguj M. (2017). What each clinical anatomist has to know about left renal vein entrapment syndrome (Nutcracker Syndrome): a review of the most important findings. BioMed Res Int.

[6] Kurklinsky A.K., Rooke T.W. (2010). Nutcracker phenomenon and nutcracker syndrome. Mayo Clin Proc.

[7] Nasa P., Jain R., Juneja D. (2021). Delphi methodology in healthcare research: How to decide its appropriateness. World J Methodol.

[8] De Mik S.M., Stubenrouch F.E., Legemate D.A. (2019). Delphi study to reach international consensus among vascular surgeons on major arterial vascular surgical complications. World J Surg.

[9] Zhang H., Li M., Jin W., San P., Xu P., Pan S. (2007). The left renal entrapment syndrome: diagnosis and treatment. Ann Vasc Surg.

[10] Black S.A., Gohel M., de Graaf R. (2024). Management of lower extremity venous outflow obstruction: results of an international Delphi Consensus. Eur J Vasc Endovasc Surg.

[11] Chen S., Zhang H., Shi H., Tian L., Jin W., Li M. (2011). Endovascular stenting for treatment of nutcracker syndrome: report of 61 cases with long-term followup. J Urol.

[12] Hartung O., Grisoli D., Boufi M. (2005). Endovascular stenting in the treatment of pelvic vein congestion caused by Nutcracker Syndrome: Lessons learned from the first five cases. J Vasc Surg.

[13] Sayed M.H., Salem M., Desai K.R., O'Sullivan G.J., Black S.A. (2022). A review of the incidence, outcome, and management of venous stent migration. J Vasc Surg Venous Lymphat Disord.

[14] Cizman Z., Zachery Paden W., Smith T., Wilhite S., Strain D., Saad W. (2024). Endovascular retrieval of a migrated covered stent from the pulmonary artery. Radiol Case Rep.

[15] Cooley C., Alexander R.A., Johns P., Fanning T., Oommen J.Z. (2023). The disappearing act: case of a migrating left renal vein stent. Oxf Med Case Reports.

[16] Luo H., Sun J., Fu J. (2023). Endovascular retrieval of a Dislocated stent in the right ventricle of a patient with nutcracker syndrome. Int Heart J.

[17] Ober M.C., Lazăr F.L., Achim A. (2022). Interventional management of a rare combination of nutcracker and Wilkie syndromes. J Pers Med.

[18] Zhang J., Li T. (2022). Stent in left renal vein stuck at the opening of tricuspid valve. Ann Thorac Surg.

[19] Miller J.S., Ramaprabhu K., Mohamed Ahmed E., Halkos M.E., Murphy D.A. (2021). Endoscopic robotic retrieval of a migrated subclavian vein stent from the right ventricle. Innovations.

[20] Sebastian T., Erdoes G., Bratu V.A., Baumgartner I., Kucher N. (2017). Endovascular extraction of a migrated large self-expanding laser-cut renal venous stent from the right ventricle. J Vasc Surg Cases Innov Tech.

[21] Wu Z., Zheng X., He Y. (2016). Stent migration after endovascular stenting in patients with nutcracker syndrome. J Vasc Surg Venous Lymphat Disord.

[22] Chen Y., Mou Y., Cheng Y., Wang H., Zheng Z. (2015). Late stent migration into the right ventricle in a patient with nutcracker syndrome. Ann Vasc Surg.

[23] Tian L., Chen S., Zhang G., Zhang H., Jin W., Li M. (2015). Extravascular stent management for migration of left renal vein endovascular stent in nutcracker syndrome. BMC Urol.

[24] Rana M.A., Oderich G.S., Bjarnason H. (2013). Endovenous removal of dislodged left renal vein stent in a patient with nutcracker syndrome. Semin Vasc Surg.

[25] Görgec B., Benedetti Cacciaguerra A., Pawlik T.M. (2022). An international expert Delphi consensus on defining textbook outcome in liver surgery (TOLS). Ann Surg.

