# Supplementary material for: Nutcracker syndrome (a Delphi consensus)
Source: J Vasc Surg Venous Lymphat Disord. 2024 Oct 2;13(1):101970. doi: 10.1016/j.jvsv.2024.101970 (PMC11764206; doi:10.1016/j.jvsv.2024.101970)
Supplement: Supplementary Table I (online only) [file mmc1.docx]

| **Supplementary: Table SI** Comments provided by reviewers after Round 1 |
| --- |
| 1. Diagnosis |
| *1.1 Symptoms and clinical features* |
| Hematuria specific to NCS is caused by the rupture of the thin-walled septum between small veins and collecting system of renal fornix. |
| *Also, macroscopic hematuria may occur due to renal collateral varicose veins around the pelvis of kidney rupturing in to the pelvi-calyceal system.* |
| *Appears to be the hypothesis we have available.* |
| Proteinuria specific to NCS is caused by increased pressure in the LRV, which leads to the release of angiotensin II and norepinephrine. |
| *Renal vein hypertension leads to release of AT II and NA, induced by changes in renal hemodynamics during prolonged standing, exercise and increase in intra-abdominal pressures.* |
| *I'm not aware of any studies that elucidated the mechanism of proteinuria in Nutcracker syndrome.* |
| NCS is not related to pelvic venous disorders. |
| *NCS can be the cause of a distended Left Gonadal Vein. However, pelvic symptoms should be investigated separately as NCS may be an incidental association.* |
| *I think they can be related, but one almost never can know for sure.* |
| *NCS is often related to PVD because of gonadic vein insufficiency which can be caused by NCS, and directly provide pelvic venous congestion syndrome.* |
| *1.2 Diagnostics* |
| A significant pressure gradient is defined as >2mm Hg. |
| *Need to indicate what position the patient is in and clarify that you intend "mean pressures".* |
| 3 Follow-up |
| *3.1 Anticoagulation or antiplatelet medication* |
| After LRV transposition, patients should be treated with platelet aggregation inhibitors for the total duration of at least 6 months. |
| *I think anticoagulation therapy would be a better option to prevent venous thrombosis.* |
| Patients must be followed up using DUS yearly. |
| *I don’t think changing severity of stenosis would be the determining factor in clinical decision.* |
| *CT-scan first.* |
| *No timing protocol has been validated.* |
| First moment of follow-up should be before 6 weeks. |
| *Clarify what kind of follow-up (CT-scan or DUS).* |
| Patients with NCS should be followed up yearly. |
| *Clarify if you are talking about post-procedure follow-up.* |
| Follow up can be ended after a period of 2 years. |
| *Clarify if you are talking about post-procedure follow-up.* |
| *Follow up should be longer because no one know when recurrence will occur.* |
