# Supplementary material for: Nutcracker syndrome (a Delphi consensus)
Source: J Vasc Surg Venous Lymphat Disord. 2024 Oct 2;13(1):101970. doi: 10.1016/j.jvsv.2024.101970 (PMC11764206; doi:10.1016/j.jvsv.2024.101970)
Supplement: Appendices [file mmc2.docx]

**Appendix A: Questionnaire sent out in first round of Delphi consensus process**

# 1. Diagnosis

### 1.1 Symptoms and clinical features

1. Nutcracker Syndrome (NCS) is a combination of clinical signs and symptoms caused by stenosis of the left renal vein (LRV).

- Strongly agree
- Agree
- Neutral
- Disagree
- Strongly disagree

1. The characteristic clinical signs and symptoms of NCS include hematuria and flank pain

- Strongly agree
- Agree
- Neutral
- Disagree
- Strongly disagree

1. The discovery of asymptomatic compression of the left renal vein (LRV) is called nutcracker phenomenon.

- Strongly agree
- Agree
- Neutral
- Disagree
- Strongly disagree

1. Hematuria specific to Nutcracker Syndrome is caused by rupture of the thin-walled septum between small veins and collecting system of renal fornix.

- Strongly agree
- Agree
- Neutral
- Disagree
- Strongly disagree

1. Proteinuria specific to Nutcracker syndrome is caused by increased pressure in the LRV, which leads to the release of angiotensin II and norepinephrine.

- Strongly agree
- Agree
- Neutral
- Disagree
- Strongly disagree

1. NCS is not related to pelvic venous disorders.

- Strongly agree
- Agree
- Neutral
- Disagree
- Strongly disagree

1. Pelvic venous disorders can be caused by NCS.

- Strongly agree
- Agree
- Neutral
- Disagree
- Strongly disagree

### 1.2 Diagnostics

1. Imaging is obligated to confirm NCS.

- Strongly agree
- Agree
- Neutral
- Disagree
- Strongly disagree

1. Doppler Ultrasonography (DUS) should be used in the diagnostic work up of NCS patients.

- Strongly agree
- Agree
- Neutral
- Disagree
- Strongly disagree

1. Because of the highly variability index and range of values of DUS in NCS, it should not be used as a single diagnostic modality to diagnose NCS.

- Strongly agree
- Agree
- Neutral
- Disagree
- Strongly disagree

1. DUS in NCS patients should at least describe the peak systolic velocity at stenosis and in the distal renal vein and the diameter of the LRV at the stenosis and distal to the stenosis.

- Strongly agree
- Agree
- Neutral
- Disagree
- Strongly disagree

1. Venous Computed Tomography (CTV) or Magnetic resonance Imaging (MRI) can demonstrate compression of the LRV between the superior mesenteric artery (SMA) and the aorta or between the aorta and the spina.

- Strongly agree
- Agree
- Neutral
- Disagree
- Strongly disagree

1. CT or MRI do not have a role in the diagnostic work up in NCS patients.

- Strongly agree
- Agree
- Neutral
- Disagree
- Strongly disagree

1. CT or MRI should always be performed before surgery to exclude other potential causes of symptoms and for accurate surgical planning.

- Strongly agree
- Agree
- Neutral
- Disagree
- Strongly disagree

1. Contrast phlebography is considered the gold standard for diagnosis of NCS.

- Strongly agree
- Agree
- Neutral
- Disagree
- Strongly disagree

1. Venous pressure measurements and observation of collateral flow are the most useful phlebographic signs of significant LRV obstruction.

- Strongly agree
- Agree
- Neutral
- Disagree
- Strongly disagree

1. The absence of a significant pressure gradient does not exclude nutcracker syndrome.

- Strongly agree
- Agree
- Neutral
- Disagree
- Strongly disagree

1. A significant pressure gradient is defined as >2mm Hg.

- Strongly agree
- Agree
- Neutral
- Disagree
- Strongly disagree

1. Intravascular ultrasound can be used to assess the severity of LRV stenosis in NCS patients.

- Strongly agree
- Agree
- Neutral
- Disagree
- Strongly disagree

# 2. Management

### 2.1 Conservative treatment

1. A conservative approach should be the first treatment option in all NCS patients.

- Strongly agree
- Agree
- Neutral
- Disagree
- Strongly disagree

1. Conservative treatment with an emphasis of weight gain should be the first step in treating all NCS patients with a low body weight (BMI under 18.5 kg/m^2^).

- Strongly agree
- Agree
- Neutral
- Disagree
- Strongly disagree

1. Aspirin should be considered if the patient with NCS shows sign of kidney failure to improve renal perfusion.

- Strongly agree
- Agree
- Neutral
- Disagree
- Strongly disagree

1. Aspirin should be prescribed when diagnosing NCS

- Strongly agree
- Agree
- Neutral
- Disagree
- Strongly disagree

### 2.2 Operative treatment

1. First choice of operative treatment is LRV transposition.

- Strongly agree
- Agree
- Neutral
- Disagree
- Strongly disagree

1. If successful LRV transposition does not lead to symptom release, the diagnosis NCS should be reconsidered.

- Strongly agree
- Agree
- Neutral
- Disagree
- Strongly disagree

1. Renal autotransplantation is an effective procedure.

- Strongly agree
- Agree
- Neutral
- Disagree
- Strongly disagree

1. Left gonadal vein (LGV) transposition can be an alternative for LRV transposition in selected patients.

- Strongly agree
- Agree
- Neutral
- Disagree
- Strongly disagree

1. LGV transposition can be performed into the left iliac vein in patients who do not have May-Thurner syndrome.

- Strongly agree
- Agree
- Neutral
- Disagree
- Strongly disagree

1. Too little evidence is present to have a preference for laparoscopic or open surgical procedures.

- Strongly agree
- Agree
- Neutral
- Disagree
- Strongly disagree

### 2.3 Endovascular treatment

1. The risk of stent migration outweighs the advantages of a percutaneous procedure, thus stenting is not recommended as a primary treatment for NCS.

- Strongly agree
- Agree
- Neutral
- Disagree
- Strongly disagree

1. With an unknown long-term outcome of renal vein stents, open interventions are a safer option for NCS.

- Strongly agree
- Agree
- Neutral
- Disagree
- Strongly disagree

1. Endovascular stenting (EVS) is the preferred treatment over open procedures.

- Strongly agree
- Agree
- Neutral
- Disagree
- Strongly disagree

# 3 Follow-up

### 3.1 Anticoagulation or antiplatelet medication

1. After LRV transposition, patients should be treated with platelet aggregation inhibitors for the total duration of at least six months.

- Strongly agree
- Agree
- Neutral
- Disagree
- Strongly disagree

1. Patients must be followed up using DUS yearly.

- Strongly agree
- Agree
- Neutral
- Disagree
- Strongly disagree

1. First moment of follow-up should be before 6 weeks.

- Strongly agree
- Agree
- Neutral
- Disagree
- Strongly disagree

1. Patients with NCS should be followed up yearly.

- Strongly agree
- Agree
- Neutral
- Disagree
- Strongly disagree

1. Follow up can be ended after a period of 2 years.

- Strongly agree
- Agree
- Neutral
- Disagree
- Strongly disagree

**Appendix B: Questionnaire sent out in round two of Delphi consensus process**

# 1. Diagnosis

### 1.2 Diagnostics

1. Contrast phlebography is considered the gold standard for diagnosis of NCS.

- Strongly agree
- Agree
- Neutral
- Disagree
- Strongly disagree

1. A significant pressure gradient is defined as >2mm Hg.

- Strongly agree
- Agree
- Neutral
- Disagree
- Strongly disagree

# 2. Management

### 2.1 Conservative treatment

1. Aspirin should be considered if the patient with NCS shows sign of kidney failure to improve renal perfusion.

- Strongly agree
- Agree
- Neutral
- Disagree
- Strongly disagree

1. Aspirin should be prescribed when diagnosing NCS

- Strongly agree
- Agree
- Neutral
- Disagree
- Strongly disagree

### 2.2 Operative treatment

1. Renal autotransplantation is an effective procedure.

- Strongly agree
- Agree
- Neutral
- Disagree
- Strongly disagree

# 3 Follow-up

### 3.1 Anticoagulation or antiplatelet medication

1. After LRV transposition patients should be treated with some form of platelet aggregation or anticoagulants.

- Strongly agree
- Agree
- Neutral
- Disagree
- Strongly disagree

1. Patients can be followed using DUS yearly in long term follow up.

- Strongly agree
- Agree
- Neutral
- Disagree
- Strongly disagree

1. First moment of imaging (CT-scan or DUS) after surgery should be before 6 weeks.

- Strongly agree
- Agree
- Neutral
- Disagree
- Strongly disagree

1. Patients with NCS who underwent surgery should be followed up yearly.

- Strongly agree
- Agree
- Neutral
- Disagree
- Strongly disagree

1. Follow up after surgery can be ended after a period of 5 years.

- Strongly agree
- Agree
- Neutral
- Disagree
- Strongly disagree

**Appendix C: Questionnaire sent out in third round of Delphi consensus process**

# 1. Diagnosis

### 1.1 Symptoms and clinical features

1. The compressive process causes varying levels of extrinsic stenosis of the renal branch, which results in asymptomatic episodes alternated with symptomatic episodes.

- Strongly agree
- Agree
- Neither agree nor disagree
- Disagree
- Strongly disagree

1. Classify these signs and symptoms concerning NCS from most to least relevant: flank pain, hematuria, proteinuria, pelvic pain.
2. Flank pain
3. Hematuria
4. Pelvic pain
5. Proteinuria
6. In case of NCS, signs and symptoms (such as hematuria and flank pain) must last >6 months.
   - - Strongly agree
     - Agree
     - Neither agree nor disagree
     - Disagree
     - Strongly disagree

### 1.2 Diagnostics

1. There is no gold standard for imaging used in diagnosing NCS.

- Strongly agree
- Agree
- Neither agree nor disagree
- Disagree
- Strongly disagree

1. The percentage of stenosis of LRV is a useful factor and >50 percent stenosis measured by duplex should be considered significant.
   - - Strongly agree
     - Agree
     - Neither agree nor disagree
     - Disagree
     - Strongly disagree
2. A distance between the AMS and aorta of <8 mm is considered abnormal.
   - - Strongly agree
     - Agree
     - Neither agree nor disagree
     - Disagree
     - Strongly disagree
3. During ultrasound; a ratio of > 4 between the diameter of the hilar renal vein and the diameter of the renal vein at the aortic mesenteric window is considered abnormal.
   - - Strongly agree
     - Agree
     - Neither agree nor disagree
     - Disagree
     - Strongly disagree
4. An aortic – SMA angle of <30 degrees is an abnormal finding.
   - - Strongly agree
     - Agree
     - Neither agree nor disagree
     - Disagree
     - Strongly disagree
5. Phlebography has a place in the diagnostic process, as it provides information about the pressure gradient, collateral veins, flow pattern and LGV.
   - - Strongly agree
     - Agree
     - Neither agree nor disagree
     - Disagree
     - Strongly disagree
6. NCS can be excluded without venography.
   - - Strongly agree
     - Agree
     - Neither agree nor disagree
     - Disagree
     - Strongly disagree
7. In case of a BMI >25 km/m2, a diagnosis other than NCS must be considered.
   - - Strongly agree
     - Agree
     - Neither agree nor disagree
     - Disagree
     - Strongly disagree
8. At least one form of cross-sectional imaging associated to one functional imaging modality should be performed in the diagnostic work-up of NCS.
   - - Strongly agree
     - Agree
     - Neither agree nor disagree
     - Disagree
     - Strongly disagree

# 3 Follow-up

1. During follow-up, patients with NCS should be checked for stent patency or severity of stenosis in LRV.
   - - Strongly agree
     - Agree
     - Neither agree nor disagree
     - Disagree
     - Strongly disagree
2. > 50 percent re-stenosis in the LRV should lead to reintervention.
   - - Strongly agree
     - Agree
     - Neither agree nor disagree
     - Disagree
     - Strongly disagree
